# Effect of selected feed additives to improve growth and health of dairy calves

**DOI:** 10.1371/journal.pone.0216066

**Published:** 2019-05-03

**Authors:** Luisa F. L. Salazar, Luis A. Nero, Maria E. M. Campos-Galvão, Cristina S. Cortinhas, Tiago S. Acedo, Luis F. M. Tamassia, Karina C. Busato, Válber C. Morais, Polyana P. Rotta, Alex L. Silva, Marcos I. Marcondes

**Affiliations:** 1 Department of Animal Science, Universidade Federal de Viçosa, Viçosa, Minas Gerais, Brazil; 2 Department of Veterinary, Universidade Federal de Viçosa, Viçosa, Minas Gerais, Brazil; 3 Department of Innovation and Applied Science, DSM Produtos Nutricionais Brasil S.A., São Paulo, São Paulo, Brazil; 4 Department of Animal Production, Institute of Animal Science, Universidade Federal Rural do Rio de Janeiro, Seropédica, Rio de Janeiro, Brazil; University of Illinois, UNITED STATES

## Abstract

The aim was to evaluate the effect of different feed additives on intake, performance, and fecal consistency index (FCI) of dairy calves from 6–60 d of age and its residual effect 15 d after weaning. Fifty Holstein calves (38 ± 1.0 kg BW) were fed 5 L/d of milk plus starter feed until weaning, and corn silage and concentrate after weaning. The treatments were: control (CON), monensin (MON; 30 mg/kg of starter), probiotic *E*. *faecium* (PROB; 70 mg/kg of starter), essential oils (EO; 300 mg/kg of starter), or PROB + EO (EOPROB). Fecal score and dry matter intake (DMI) were measured daily, and animals were weighed every 15 d. A DNA extraction from feces was performed to identify the presence of microorganisms (*E*. *coli*, *Hafnia*, *Shiguella*, *Lactobacillus spp*, *Enterococcus spp*, and *Enterococcus faecium* NCIMB 10415) by PCR. Two 72-h digestibility trials were performed at days 20–28 and 50–56, by total fecal collection. The DMI before weaning was greater for EO (903.0 g/d) compared with MON (794.3 g/d) and EOPROB (783.1 g/d). The FCI decreased during pre-weaning for EO and MON. Average daily gain (ADG) and feed efficiency (FE) did not differ among treatments before weaning. After weaning, DMI and FCI did not differ among treatments. The EO had greater ADG (917.5 g/d) compared with CON (615.8 g/d) and PROB (592.6 g/d). The FE improved with EO (0.72 g/g) over CON (0.36 g/g), MON (0.49 g/g), and PROB (0.36 g/g). The PCR results showed absence of *E*. *faecium* NCIMB 10415 in animals fed PROB and CON. Animals fed PROB had greater intake of CP and NDF than animals fed EOPROB. The EO can be added to the dairy calf ration to improve fecal score and increase DMI. The pre-weaning FCI decrease with MON and increase with PROB.

## Introduction

During the first days of life, a calf’s rumen and microbial population are not completely developed and functional, and stress factors like dehorning, weaning, vaccination, or extreme changes of temperature may cause decreased immunity, resulting in diarrhea, then weight loss or reduced performance [1]. Farmers often use antibiotics for prevention and treatment of diarrhea in calves. There has been great concern worldwide about how this practice might impact food security, since it is possible to observe residual medications in foods of animal origin, affecting human health in the form of allergies and intoxication, and inducing antimicrobial resistance in bacteria both in animals and in humans [2,3].‬‬‬‬‬‬‬‬‬‬‬‬‬‬‬‬‬‬‬‬‬‬‬‬‬‬‬‬‬‬‬‬‬‬‬‬‬‬‬‬‬‬‬‬‬‬‬‬‬‬‬‬‬‬‬‬‬‬

Monensin is an ionophore antibiotic that exhibits anticoccidial and antibacterial properties. It is commoly used, commercially, as a coccidiostat for poultry and as a growth promoter for ruminants [4]. However, the use of ionophores in livestock production has been banned in several countries due to development of monensin resistance by some bacteria [5]. Consequently, some products such as probiotics and essential oils have been used as alternatives to antibiotics, aiming minimize environmental risks and ensure food safety [3,6].

Probiotics have beneficial effects on the gastrointestinal tract, for instance via modulation of the immune system and decrease the incidence of diarrhea [7]; however, the results are variable. Probiotic strains of *Enterococcus faecium* have been studied extensively in piglets, and shown positive impact on intestinal microbiota, reflected in the reduction of enteropathogenic bacterial load of suckling piglets fed *E*. *faecium* SF68 and in the presence of the probiotic in feces during the whole period of supplementation [8]. Although the benefits of probiotics have been described for dairy cattle [9,10], there is limited data in the literature on the effects of *Enterococcus faecium* NCIMB 10415, especially on dairy calves [11].

Essential oils (EO) have been widely used as a new class of feed additive to improve the intestinal microbiota of domestic animals and have positive effects on calves performance and healthy [3,6]. The EO are a mixture of many chemical compounds, mainly terpenes and terpene derivatives, [12] that have antimicrobial activity [13]. Generally, EO are more effective against Gram-positive than Gram-negative bacteria since the structure of Gram-positive bacteria cell wall allows hydrophobic molecules to easily penetrate the cell and to act both on the cell wall and within the cytoplasm, while Gram-negative bacteria are made more resistant by the lipopolysaccharidesin their outer membrane [14]. However, carvacrol, eugenol, and thymol are capable of disintegrating the outer membrane of Gram-negative bacteria such as *Escherichia coli* and *Salmonella Typhimurium* [15]. In addition, it has been suggested that adding EO to the concentrate can increase dry matter intake (DMI) and average daily gain (ADG) of Holstein calves [16].

We hypothesized that the addition of probiotics, EO or monensin, will improve the performance and intestinal environment of calves, reducing the incidence of diarrhea. We also hypothesized that the combined use of EO and probiotics will increase the beneficial aspects of these additives relative to when they are used separatelly. The objective was to evaluate the effect of monensin, EO, probiotic *Enterococcus faecium* NCIMB 10415, or the combination of EO and probiotic on intake, performance, diarrhea incidence, and fecal microbial population of suckling calves from 6 to 60 days of age, and their residual effect 15 days after weaning.

## Materials and methods

The experiment was carried out at the Department of Animal Science of the Universidade Federal de Viçosa-UFV (Viçosa, MG, Brazil), and it was approved by the Ethics Committee in the Use of Production Animals of the UFV, process No. 26/2017.

### Animals, feeding, and treatments

Fifty (26 female and 24 male) six-day-old Holstein calves, from the herd of Universidade Federal de Viçosa-UFV (Viçosa, MG, Brazil) and with an average initial body weight of 38 ± 1.0 kg, were fed 5 L/day of raw milk that was delivered twice per day (2.5 L at 07:00 h and 2.5 L at 15:30 h). Calves were given *ad libitum* access to starter feed formulated with 60% whole ground corn, 20% soybean meal, and 20% a commercial mineral and vitamin premix, containing the following treatments: control (CON), without feed additives; probiotic *Enterococcus faecium* NCIMB 10415 (PROB, 70 mg/kg of starter feed, CFU/kg 1.4E+09, Cylactin, DSM Nutritional Products, Brazil); EO (300 mg/kg of starter feed; blend of thymol, guaiacol, eugenol, vanilin, salicylaldehyde, and limonene, Crina Ruminants, DSM Nutritional Products, Brazil); probiotic plus essential oils (EOPROB; treatments PROB+EO), and monensin (MON, 30 mg/kg of starter feed, Rumensin, Elanco, Brazil). The feed additives were previously mixed into the commercial mineral and vitamin premix, according to each treatment and then mixed to ground corn and soybean meal to perform the final starter feed.

The animals were supplementated from the 6th day of life to weaning (60 days of life). The animals were weaned abruptly and after that, all animals were fed the control treatment plus corn silage (which was added to the diet imediately after weaning) *ad libitum* separately for 15 more days. The starter and clean water were offered in separate buckets in the morning. After the end of this study the animals were re-incorporated to the herd of Universidade Federal de Viçosa to be used in future studies (Table 1).

**Table 1:** Chemical composition of starter feed, milk, and sorn silage used in the present study.

Item^a^	Milk	Starter feed^b^	Corn silage
**Chemical composition (g/kg)**			
**Dry matter**^a^	112.4	903.2	253.0
**Organic matter**	926.2	930.7	942.0
**Crude protein**	256.7	173.2	85.6
**Ether extract**	285.6	47.0	28.5
**Neutral detergent fiber**	-	172.5	514.0
**Non-fibrous carbohydrates**	407.5	538.0	313.9

^a^As fed basis.

^b^Composed by 60% of whole ground corn, 20% of soybean meal, and 20% of a commercial mineral and vitamin premix.

### Measures of intake, weight, fecal score, and digestibility trial

The intake of starter feed and silage (post-weaning) was calculated every morning, as the difference between the amount offered and leftovers. Concerning milk intake, the animals ingested all milk provided to them. The animals were weighed and measured at withers height (WH) and at croup height (CH; measurements taken across the hip bones) at the beginning of the experiment, followed by 15 days interval, at weaning, and at the end of the experiment.

The animals' fecal consistency score was monitored daily (scored from 1 to 4, where 1 = pasty, 2 = pulpy, 3 = soupy, 4 = watery) [17]. Calves with fecal consistency 3 or 4 were classified as having diarrhea. When the animal presented pale and dry mucous membranes along with diarrhea, an intervention with hydration was delivered: 8 g of NaCl, 8 g of NaHCO_3_, 2g of KCl, 15g of dextrose, and 2 L of warm water. The fecal consistency index (FCI) was calculated according to the following equation [18]:

$$\mathrm{FCI}=\frac{\left[\left(\mathrm{dS}1\times 1)+(\mathrm{dS}2\times 2)+(\mathrm{dS}3\times 3)+(\mathrm{dS}4\times 4\right)\right]}{\mathrm{Td}\times 4}\times 100$$ (1)

where: FCI = fecal consistency index; dS1, dS2, dS3, dS4 = number of days with fecal consistency of score 1, 2, 3, 4, respectively; and Td = total days.

Two 72-h digestibility trials were performed at days 26–28 (period 1) and 54–56 (period 2), with total fecal collection. Initially, five animals per treatment started the digestilbity trials; however, after the first day of collection, some animals showed high levels of stress and diarrhea, likely due to high tempeatures, humidity and stress caused by the digestiblity trial itself. Therefore, these animals were removed from the digestibility trial results, and data from only three animais per treatment were used. At the end of each day of feces collection, total feces per calf were weighed, homogenized, and sampled.

### Chemical analysis

Samples of feces were oven-dried (55°C) for 72 h and then ground to 1 mm. Samples of milk were freeze-dried and ground to 1 mm in a knife mill [19]. Samples of feeds and feces were evaluated for dry matter (DM; [20]; method 934.01), crude protein (CP; [20]; method 990.13), ash ([20]; method 942.05), and neutral detergent fiber (NDF; [19]; INCT-CA method F-002/1). Ether extract (EE) was evaluated according to AOCS [21] and non-fibrous carbohydrate (NFC) was calculated as follows:

NFC=1000‑(CP+NDF+EE+ash) (2)

where: NFC = non-fibrous carbohydrate, CP = crude protein, NDF = neutral fiber detergent, and EE = ether extract. All values are given in g/kg.

### DNA extraction and PCR from fecal samples

Before the morning feeding on days 5, 30, 60 (weaning day), and 75 (last day of experiment), fecal samples of all animals (approximately 50 g) were collected directly from the rectum. Each sample was homogenized with a sterilized spatula, then between 180 and 220 mg of the homogenized sample was placed in an eppendorf tube and stored in an ultra-freezer at -80°C.

Extraction of DNA was performed using the QIAamp DNA Stool Mini Kit (Qiagen, Hilden, Germany) following the manufacturer's recommendations. After extracting the DNA, 2 μL of each sample was analyzed in NanoDrop Lite (Thermo Fisher Scientific, Waltham, Massachusetts, USA) to perform DNA quantification. After these procedures, the samples were stored at -20°C for subsequent analyses.

Conventional PCR was performed in the Molecular Biology Laboratory (BIOMOL) at Universidade Federal de Viçosa (UFV), to identify the presence of *E*. *coli*, *Hafnia*, *and Shiguella* (ENTERO), *Lactobacillus spp* (LAC), *Enterococcus spp* (ENT), and *E*. *faecium* NCIMB 10415 in feces using species-specific PCR primers according to Starke et al. [8] as shown in S1 Table. This data was used to calculate the frequency of fecal presence of each microrganism.

Amplification reactions were conducted in a total volume of 25 μL containing: 12.5 μL of GoTaq Green Master Mix 2X (Promega Corporation, Madison, Wisconsin, EUA), with 0.5 μL of each primer (10 mM/μL) and 9.5 μL of nuclease-free water (S1 Table). Reactions were performed under the following conditions. Enterobacter and *Lactobacillus*: initial denaturation at 95°C for 2 minutes, followed by 35 cycles of denaturation at 94°C for 30 seconds, annealing at 58°C for 30 seconds, extension of 72°C per 1 minute, and final extension of 72°C for 5 minutes. *Enterococcus spp* and *E*. *faecium* NCIMB 10415: initial denaturation at 95°C for 2 minutes, followed by 35 cycles of denaturation at 94°C for 30 seconds, annealing at 60°C for 30 seconds, extension of 72°C per 1 minute, and final extension of 72°C for 5 minutes. The PCR products were electrophoresed on a 1.5% agarose gel in Tris/borate/EDTA buffer. The products were visualized by staining with UniSafe Dye (0.5 μg/mL; Uniscience Corporation, São Paulo, Brazil).

### Statistical analysis

The data on performance, intake, FCI, and feed efficiency (FE) were analyzed according to a completely randomized block design, with the animals being blocked by gender:

$$Y_{ijk}=\mu +{FA}_{i}+\beta_{j}+{\left(FA\times \beta \right)}_{ij}+\varepsilon_{ijk}$$

where Y_ijk_ = dependent variable, μ = overall mean; FA_i_ = fixed effect of feed additives, β_j_ = random effect of block (gender), FA × β_ij_ = random effect of interaction between feed additives and block (gender), and ε_ijk_ = random error. As the interaction between feed additives and block (gender) was not significant, this effect was removed of the statistical model [22].

Data from digestibility trials (intake and digestibility) were evaluated as described above, but included the period as a repeated measure. An additional FCI analysis was carried out, including the week of life as repeated measures to verify critical diverging moments between treatments. Period (digestibility trials) or week (fecal consistency) were evaluated according to the follow model:

$$Y_{ijklm}=\mu +{FA}_{i}+\beta_{j}+{\left(FA\times \beta \right)}_{ij}+\delta_{ijk}+T_{l}+{\left(FA\times T\right)}_{il}+\varepsilon_{ijklm}$$

where Y_ijklm_ = dependent variable, μ = overall mean; FA_i_ = fixed effect of feed additives, β_j_ = random effect of block (gender), FA × β_ij_ = random effect of interaction between feed additives and block (gender), *δ*_ijk_ = random error where the variance between animals within treatments is equal to the covariance between repeated measurements within animals, T_l_ = fixed effect of sampling time; FA × T_il_ = fixed effect of interaction between feed additives and time and ε_ijklm_ = random error. The interaction between feed additives and block (gender) was not significant and was removed [22]. The variance components, compound symmetry, heterogeneous compound symmetry, heterogeneous first-order autoregressive, and unstructured matrices of (co)variance were tested. The matrix selection was based on the Corrected Akaike’s Information Criterion, and we selected heterogeneous first-order autoregressive covariance structure.

All procedures were performed using MIXED procedure of SAS [23]. For PCR data analysis, a Wald test was performed to determine difference between treatments. Comparisons between treatments were performed by comparing least square means using Student’s t-tests at *P* < 0.05.

## Results

### Intake, digestibility, and performance

Intake of DM, organic matter (OM), CP, NDF, EE, and NFC was greater (*P* < 0.05) during the second digestibility trial (50–56 days) when compared with the first trial (20–28 days; Table 2). Animals fed PROB had greater (*P* < 0.05) intake of DM and CP when compared with EOPROB and greater (*P* < 0.05) NDF intake when compared with CON and EOPROB (Table 2). The EE and OM intake was not affected (*P* > 0.05) by the treatments. The NFC intake was greater (*P* = 0.006) for animals fed EO (526.1 g/d) when compared to CON (403.9 g/d), MON (423.8 g/d), and EOPROB (307.1 g/d). Additionally, before weaning, the average DMI over the course of the experiment was greater (*P* = 0.046) for EO (903.0 g/d) compared with MON (749.3 g/d) and EOPROB (783.1 g/d; Table 3), while no difference was found between the PROB and CON treatments (843.9 and 845.0 g/d, respectively). On the other hand, there was no observed effect (*P* = 0.171) of treatments on DMI after weaning (Table 3).

**Table 2:** Intake and digestibility during two digestibility trials (1: 20–28 d; 2: 50–56 d) of dairy calves fed different additives in the starter feed (n = 15).

Item^a^	Treatment^b^					SEM^c^	Trial		SEM^c^	*P*-Value^d^		
	CON	MON	PROB	EO	EOPROB		1	2		FA	T	FA × T
**Intake, g/d**												
**DM**	839.7^a^^b^	892.4^a^^b^	1184.6^a^	1042.6^a^^b^	804.0^b^	118.08	807.5^b^	1097.8^a^	72.31	0.022	0.001	0.934
**OM**	825.7	965.3	1074.4	1011.0	767.9	88.39	813.7^b^	1044.0^a^	61.18	0.177	0.001	0.991
**CP**	193.3^a^^b^	202.5^a^^b^	254.6^a^	226.6^a^^b^	186.5^b^	20.61	187.4^b^	237.9^a^	12.34	0.022	0.001	0.912
**NDF**	61.6^b^	76.8^a^^b^	107.6^a^	62.5^a^^b^	34.9^b^	29.83	43.5^b^	93.8^a^	29.26	0.041	0.016	0.206
**EE**	180.6	180.3	181.8	182.1	173.2	3.95	173.7^b^	185.5^a^	2.73	0.571	0.001	0.987
**NFC**	403.9^b^	423.8^a^^b^	454.8^a^^b^	526.1^a^	307.1^c^	22.69	383.9^b^	462.4^a^	19.72	0.063	0.032	0.417
**Digestibility, g/kg**												
**DM**	920	930	930	950	930	13.1	930	930	7.45	0.648	0.340	0.276
**OM**	930	940	940	960	870	31.3	920	930	22.3	0.376	0.604	0.372
**CP**	910	900	920	940	930	14.2	930	910	9.34	0.627	0.452	0.381
**NDF**	526^b^	762^a^	715^a^^b^	751^a^	614^a^^b^	11.4	639	725	39.1	0.047	0.222	0.357
**EE**	970	980	970	980	950	16.5	970	970	6.8	0.377	0.906	0.421
**NFC**	965^b^	986^a^	975^a^^b^	990^a^	971^b^	4.6	977	978	3.1	0.007	0.824	0.128

^a^DM = dry matter; OM = organic matter; CP = crude protein; NDF = neutral detergent fiber; EE = ether extract; NFC = non-fibrous carbohydrates.

^b^CON = control; MON = monensina; PROB = probiotico *Enterococcus faecium* (NCIMB 10415); EO = essential oils blend; EOPROB = EO + PROB. Means in a same row with different superscripts are significantly different (*P* < 0.05).

^c^Standard error of mean.

^d^FA = fixed effect of feed additives; T = fixed effect of time (digestibility trial); FA × T = fixed effect of interaction between FA and T.

**Table 3:** Dry matter intake (over the experiment), peformance, feed efficiency and faecal score of Holstein calves fed different additives in the starter feed (n = 50).

Item^a^	Treatment^b^					SEM^c^	*P*-Value
	CON	MON	PROB	EO	EOPROB		
**Before weaning**							
**DMI, g/d**	845.1^a^^b^	749.3^b^	843.9^a^^b^	903.0^a^	783.1^b^	43.6	0.046
**ADG, g/d**	663.6	605.1	680.8	616.0	602.2	70.21	0.384
**FE, g/g**	0.77	0.76	0.82	0.68	0.75	0.081	0.319
**FCI**	0.66^a^	0.59^b^	0.71^a^	0.59^b^	0.65^a^	0.024	0.012
**WH, cm/d**	0.28	0.27	0.32	0.27	0.24	0.034	0.608
**CH, cm/d**	0.34	0.34	0.39	0.31	0.32	0.032	0.455
**After weaning**							
**DMI, g/d**	1637.5	1538.3	1590.8	1470.7	1380.7	134.46	0.171
**ADG, g/d**	615.8^b^	733.2^a^^b^	592.6^b^	917.5^a^	794.7^a^^b^	103.51	0.023
**FE, g/g**	0.36^c^	0.49^b^^c^	0.36^c^	0.62^a^	0.60^a^^b^	0.071	0.001
**FCI**	0.78	0.79	0.77	0.76	0.79	0.043	0.651
**WH, cm/d**	0.26	0.17	0.28	0.24	0.33	0.056	0.277
**CH, cm/d**	0.23	0.16	0.24	0.32	0.27	0.051	0.281

^a^DMI = dry matter intake; ADG = average daily gain; FE = feed efficiency; FCI = faecal consistency index; WH = withers height gain; CH = croup height gain.

^b^CON = control; MON = monensina; PROB = probiotico *Enterococcus faecium* (NCIMB 10415); EO = essential oils blend; EOPROB = EO + PROB. Means in a same row with different superscripts are significantly different (*P* < 0.05).

^c^Standard error of mean.

The DM, OM, CP, and EE digestibility was not different (*P* > 0.05) among treatments. However, NDF and NFC digestibilities were greater (*P* < 0.05) for MON and EO when compared to other treatments (Table 2).

The ADG, FE, WH, and CH did not differ (*P* > 0.05) among treatments before weaning (Table 3). After weaning, WH and CH were not affected (*P* > 0.05) by treatments. Calves from the EO group had a greater (*P* = 0.023) ADG (917.5 g/d) than those from the PROB and CON groups (592.6 and 615.8 g/d, respectively). The MON and EOPROB groups had similar ADG after weaning (733.2 and 794.7 g/d, respectively) compared with EO (917.5 g/d), although EO resulted in a numerical improvement of 20.1% and 13.4% in ADG compared with MON and EOPROB, respectively. The FE of EO-fed calves improved (*P* = 0.001) over CON, MON, and PROB (720, 360, 490, and 360 g/kg, respectively; Table 3).

### Fecal score

Before weaning, calves from EOPROB, PROB, and CON groups showed similar FCI (0.65, 0.71, and 0.66, respectively; Table 3), and calves fed PROB treatment had greater incidence of diarrhea. In contrast, animals fed EO and MON had similar FCI (0.59 for both), which was lower (*P* = 0.012) than other treatments, with low incidence of diarrhea.

**Fig 1:**
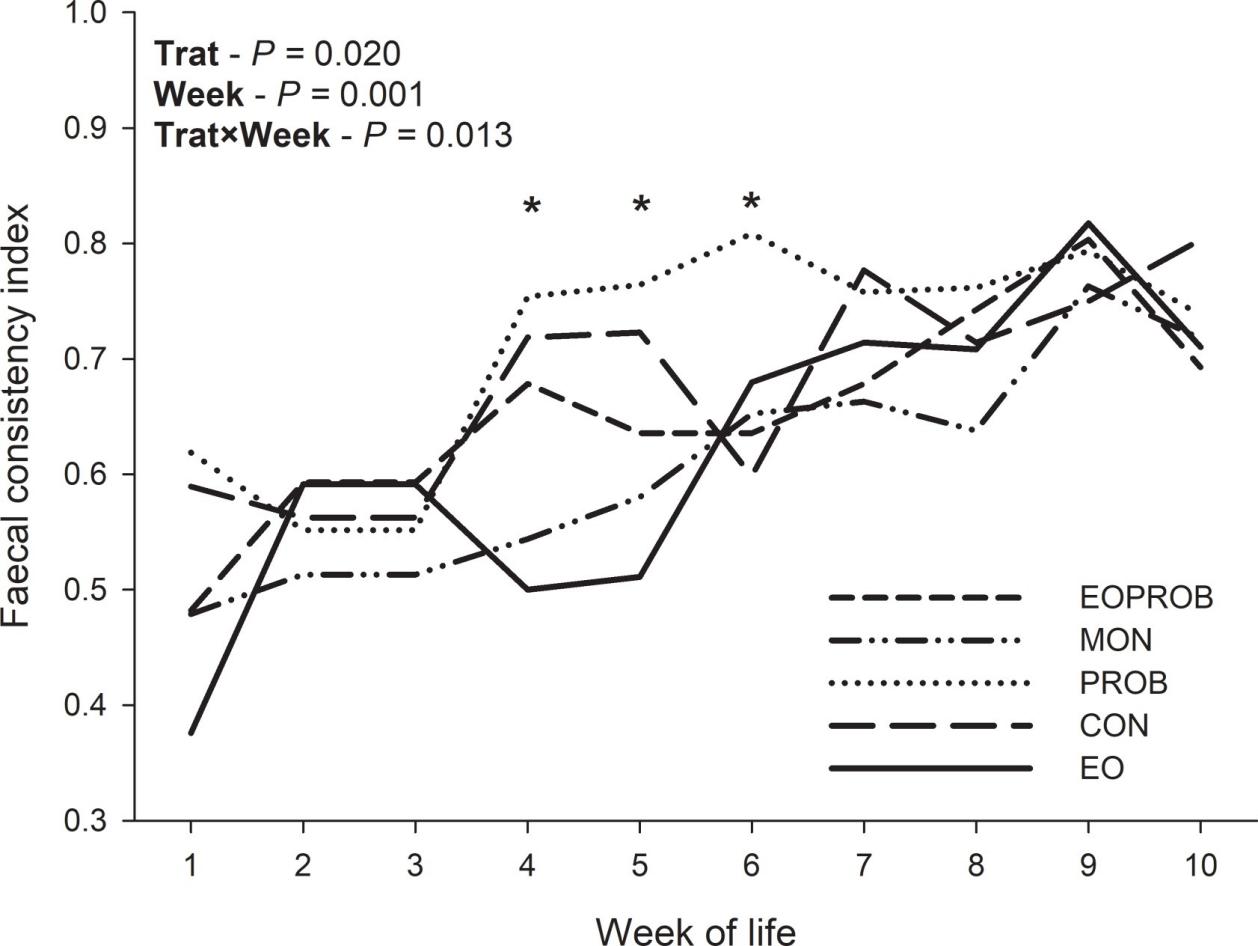
Fecal score of Holstein calves fed different additives in the concentrate: EO (Essential Oils), PROB (probiotic *Enterococcus faecium* NCIMB 10415), MON (monensina), EOPROB (EO + PROB), CON (Control). *Indicates significance (*P* < 0.05).

After weaning, FCI did not differ among treatments (*P* = 0.651; Table 3). The weekly data analysis of fecal consistency (Fig 1) revealed that the effects observed previously were mainly due to an increase in the fecal score of the PROB-fed animals and a decrease in the score in animals fed EO and MON from weeks 4 to 6 (*P* = 0.013). There was a continuous increase in the fecal scores of the animals over the weeks of life, and at week 10 there was a decrease in animal fecal scores (Fig 1).

### Fecal microorganisms

**Fig 2:**
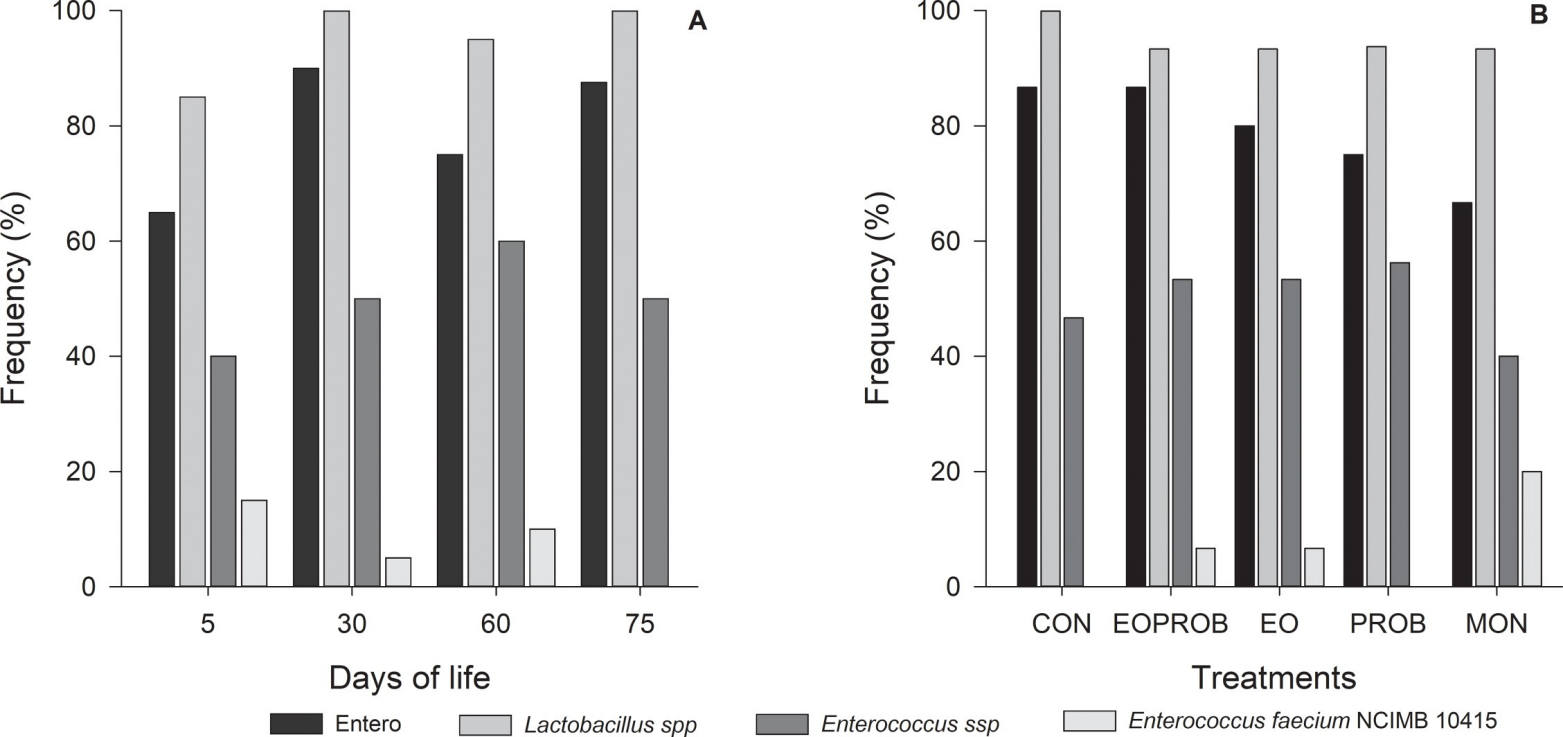
Frequency of microorganisms in feces of dairy calves, according to days of life (A) or the different additives in the concentrate (B), as follows: CON (control). EO (essential oils), PROB (probiotic *Enterococcus faecium* NCIMB 10415), MON (monensin), EOPROB (EO + PROB).

Effects of days of life (5, 30, 60, and 75), treatments, or interaction between time of feces sampling and treatment on fecal microorganisms were not observed (*P* > 0.05). However, when analyzing the frequency of fecal microorganisms using PCR results, on day 5 of life (before the beginning of supplementation) all four microorganisms were found, with a lower proportion of the probiotic strain, independent of the treatment (Fig 2A). On day 30, frequency of the microorganisms increased, except for the probiotic strain (*Enterococcus faecium* NCIMB 10415), which decreased. On day 60, the presence of ENTERO (*E*. *coli*, *Hafnia*, *and Shiguella)* and LAC (*Lactobacillus spp*) decreased slightly relative to day 30, but ENT (*Enterococcus spp*) continued to increase, and the probiotic strain increased relative to day 30. On day 75, when only the control starter was fed to all animals, there was a small increase in the frequency of ENTERO and LAC, and a decrease in ENT, whereas the probiotic strain was again not present (Fig 2A).

When the frequency of each microorganism was considered by treatment (Fig 2B), the frequency of LAC was the same for all treatments with additives and higher for CON. In feces of animals fed EO and EOPROB, the same frequency of ENT and the probiotic strain was observed, and the presence of ENTERO was only slightly lower in EOPROB when compared with EO. In feces of animals fed PROB, the observed frequency of ENTERO was lower compared with EOPROB and EO, and the presence of ENT was higher compared with other treatments (Fig 2B). In stool samples from PROB and CON treatments, the probiotic strain was not detected. In feces of animals fed MON, it was observed a lower frequency of ENTERO and ENT compared to other treatments, and the frequency of the probiotic strain was greater in the MON treatment compared to other treatments (Fig 2B).

## Discussion

### Intake, digestibility, and performance

The EO are recognized for being flavor enhancers, which are responsible for increasing intake in various animal species, including dairy calves [16,24,25]. Therefore, it is suggested that the EO blend used in this study might have a flavor effect on starter feed, which contributed to increase DMI, as was previously observed [16,24]. Besides having a flavor effect, the stimulation on intake promoted by EO can be linked to better intestinal health once the combination of thymol and cinnamaldehyde could potentially control proliferation of pathogenic bacteria and contribute to better gut health [25]. Carvacrol and thymol have been found to reduce the number of intra-epithelial lymphocytes and increase the ratio of villus height to crypt depth in the distal small intestine, also suggesting an improved gut health [26]. Therefore, improvement in gastrointestinal health as a result of EO supplementation may enhance the intestinal availability of essential nutrients for absorption, and consequently lead to better growth performance and greater feed intake [27]. Additionally, it is important to highlight that MON is recognized by having negative effects on DMI of both adult animals and suckling calves [28–30], which is in accordance with the results of the present study (Table 3). However, to the best of our knowledge, the reasons to the lower DMI presented by animals of EOPROB treatment remains unclear.

Greater NDF digestibility was expected in MON and EO-fed animals. Ionophores supplementation increases the ruminal concentration of propionate, which, in turn, causes a decrease in consumption, stimulating the sense of satiety and consequently a higher retention rate of feed in the rumen [31]. Considering that EO has a rumen mechanism of actuation similar to ionophores, mainly on Gram-positive bacteria, it can be expected a similar result on digestibility [13]. Therefore, the increase in fiber digestibility can be a result of the longer retention time of fiber in the rumen that favors microbial digestion. Nevertheless, recent studies did not find differences in volatile fatty acids concentrations or rumen pH and the ruminal rate when EO or MON were supplied [32,33]. This discrepancy among different studies may be related to factors such as diet composition, period of adaptation to the product, time of sample collection, and type and concentration of feed additives [32,33]. The increase in NFC digestibility that was observed for MON and EO is, apparently, linked to a stimulation of pancreatic amylase secretion and an increase of its activity in the small intestine, which plays a major role in carbohydrate digestion and absorption [34].

At the pre-weaning stage, ADG and FE were not different among treatments. These results concur with other studies in which probiotics or EO were fed to calves before weaning [35]. The significant increase in ADG and FE after weaning of EO-fed calves shows that there was a positive residual effect of EO 15 days after stopping supplementation with that additive. This response was also present in EOPROB-fed calves. However, as we did not observe any significant PROB effect, it is likely that EOPROB-fed animals had increased ADG and FE mainly due to an EO effect. The EO are comprised of different chemical compounds, including fatty acids such as oleic acid (18:1), linoleic acid (18:2), palmitic acid (16:0), stearic acid (18:0), and linolenic acid (18:3) [36]. It is believed that these fatty acids have anti-inflammatory effects, reducing the stress commonly observed during 15–30 days of life and during the post-weaning period, since long-chain fatty acids activate the peroxisome proliferator-activated receptors (PPARs), which are members of the nuclear-hormone-receptor superfamily and transduce a wide variety of signals, including environmental, nutritional, and inflammatory events. They act as positive acute-phase proteins capable of decreasing the inflammatory response, with long-term effects [37]. Additionally, it we suspect that the residual effect of essential oils after weaning might occur due to different GIT microbiota colonization/selection during the supplementation period. However, there are no studies in the literature supporting this speculation, what reinforce the necessity of future studies focusing on microbiota colonization/selection in pre-weaned calves fed essential oils. In contrast, the MON and PROB group had lower ADG and FE after weaning when compared with EO, and it was even lower than before weaning, suggesting that there was no residual effect in those animals.

### Fecal score

Before weaning, FCI was lower for animals fed EO and MON additives, indicating lower incidence of diarrhea and greater intestinal health [1]. The mechanism of action of EO against enteropathogenic bacteria can be explained by their typical hydrophobicity, which causes a disruption of bacterial structures that leads to increased permeability due to an inability to separate EO from the bacterial cell membrane [14]. The EO are generally most effective against Gram-positive microorganisms, since they manage to easily interact with the tetrapeptides presents in the membrane of peptidoglycans, inactivating enzymes such as transpeptidases, increasing permeability, and destroying the cell [38]. Gram-negative bacteria are more resistant, due to the lipopolysaccharides (which consist of lipid A, the core polysaccharide, and the O-side chain) contained in their outer membrane [14]. However, Stein and Kil [15] found that carvacrol, eugenol, and thymol are capable of disintegrating the outer membrane of Gram-negative bacteria such as *Escherichia coli* and *Salmonella* Typhimurium, two of the compounds present in our EO blend (thymol, guaiacol, eugenol, vanillin, salicylaldehide, and limoneno). The activity of EO or their components or both is not attributable to a single event because changes in molecular structures, e.g., the hydroxyl group (OH^−^), can enhance antibacterial activity of some terpenes [13]. The fact that five of six (timol, guaiacol, eugenol, vanillin, and salicylaldehide) EO present in our blend contain a OH^−^ group could explain its strong antimicrobial activity and the reduction of diarrhea in EO-fed calves.

Monensin, on the other hand, acts against pathogenic bacteria by facilitating ion transport across the bacterial cytoplasmic membrane. It does this via the formation of liposoluble complexes with a hydrophobic exterior and a hydrophilic interior able to bind sodium and potassium cations. The result is increased permeability of the cellular membranes to such ions, promotion of an osmotic imbalance, increased energy expenditure, and subsequent cell death. This mechanism leads to improvements in animal performance and lower incidence of diarrhea.

The ability of probiotic strains to hydrolyze bile salts has often been included in the selection criteria for probiotic strains. Several bile salt hydrolases have been identified and characterized, and bile salt hydrolase activity has been detected in *Enterococcus faecium* [39]. However, bile salt hydrolase activity may be a colonization factor favoring intestinal growth, as suggested by Moser and Savage [40], and it could be viewed as a potential virulence factor, especially in enterococcus strains that carry other recognized virulence traits [39]. Many bacteria are able to deconjugate bile salts by a specific hydrolase; this mechanism produces a reduction in cholesterol absorption at the intestinal level, leading to increased cholesterol in the feces and a higher passage rate [41]. However, there was no relationship between occurrence or severity of diarrhea and performance of PROB-fed animals. Thus, the positive effect of probiotics on growth performance of calves may only be present when their health status is compromised [42]. Additionally, it is important to highlight that the use of the probiotics mixed to the liquid feed, instead in the starter feed, could improve its utilization and effects in the gut, once that probiotics’ rumen degradation could be reduced.

The weekly analysis of fecal consistency showed an increase in FCI from week 4 in the PROB-fed animals, and lower FCI for EO- and MON-fed animals. At day 35, all animals were dehorned, a very stressful procedure, but we found that this procedure was less likely to affect the health of animals fed EO and MON, compared with PROB-fed calves. After week 6, the fecal score for all animals increased significantly, which may have been due to the stress of the digestibility trial performed between weeks 6 and 7, and to post-weaning stress at week 8. The fecal score started to decrease for all animals after week 9, indicating that animals adapted to the enviroment.

### Fecal microorganisms

We did not detect the probiotic PROB in those animals fed the same probiotic or in those given the control treatment. This result suggests that the probiotic was not able to survive in the gastrointestinal tract of the calves. In order to survive in the gastrointestinal tract, bacteria need to adhere to the intestinal wall (or develop faster than the speed of peristalsis) and to reach and colonize the intestine, which requires that they be resistant to an acidic pH and bile acids [43]. The PROB-fed animals had a higher FCI, with higher incidence of diarrhea and no effect on nutrient digestibility. This supports the hypothesis that the probiotic bacteria did not survive and that there was therefore no positive effect for the animals in this treatment.

Probiotic bacteria (*Enterococcus faecium* NCIMB 10415) were detected in animals fed MON, EO, and EOPROB (Fig 2B), which could be explained by the ability of MON and EO to promote increased growth of beneficial bacteria. Since *E*. *faecium* is naturally present in the gatrointestinal tract of calves, being found mainly in saliva, and small intestine [44], these treatments may have promoted the growth of this bacterial species, specifically the strain included in this study. This may also explain why the probiotic strain did not appear in feces collected at day 75, when the calves did not receive the additives. Finally, the PROB treatment was similar to the CON treatment that did not include probiotic bacteria, while the EO treatment showed a similar result to the EOPROB treatment. Together these findings strongly support the hypothesis that the probiotic was not present in the gastrointestinal tract of those animals.

## Conclusions

The EO proved to be a good alternative for improving the health of calves, as it decreased the incidence of diarrhea. In addition, EO facilitated greater DMI and improved digestibility, and had residual positive post-weaning effects on ADG and FE. Monensin improved the health of calves, decreasing the incidence of diarrhea in the pre-weaning period. The probiotic *E*. *faecium* NCIMB 10415 did not show positive results in fecal score evaluation, ADG, or digestibility, as it did not appear to survive in the gastrointestinal tract of calves. Additionally, it is important to emphasize that further studies are necessary to clarify some of the results found in this study, as well as, to evaluate these treatments under different environmental conditions.

## Supporting information

S1 Table Primer sequence, product length (base pairs-bp), and annealing temperature (°C) used for PCR in calves receiving mineral supplementation with monensin, probiotic *Enterococcus faecium* NCIMB 10415, essential oils, probiotic + essential oils or mineral control. (DOCX)

S1 File Dataset used in the present study. (XLSX)
