# Supplementary material for: Effect of selected feed additives to improve growth and health of dairy calves
Source: PLoS One. 2019 May 3;14(5):e0216066. doi: 10.1371/journal.pone.0216066 (PMC6499422; doi:10.1371/journal.pone.0216066)
Supplement: S1 Table — (DOCX) [file pone.0216066.s001.docx]

**S1 Table. Primer sequence, product length (base pairs-bp), and annealing temperature (°C) used for PCR in calves receiving mineral supplementation with monensin, probiotic *Enterococcus faecium* NCIMB 10415, essential oils, probiotic + essential oils or mineral control.**

| Specificity (Target name) | Primer name | Sequence | Product length[bp] | Annealing temperature [°C] |
| --- | --- | --- | --- | --- |
| *E. coli/ Hafnia/ Shiguella (16S rRNA)* | Entero-F | GTTAATACCTTTGCTCATTGA | 340 | 58 |
|  | Entero-R | ACCAGGGTATCTAATCCTGTT |  |  |
| *Lactobacillus spp. (16S rRNA)* | LAC-1 | AGCAGTAGGGAATCTTCCA | 341 | 58 |
|  | LAC-2 | CACCGCTACACATGGAG |  |  |
| *Enterococcus spp. (16S rRNA)* | Ent1 | CCCTTATTGTTAGTTGCCATCATT | 144 | 61 |
|  | Ent2 | ACTCGTTGTACTTCCCATTGT |  |  |
| *E. Faecium NCIMB10415 (plasmid)* | Cyl-1a | TCGGAATTTGCCAGAGAGAC | 208 | 60 |
